# Asymmetric distribution of cerebral microbleeds in patient with infarction and atrophy: A case report

**DOI:** 10.1016/j.radcr.2026.03.037

**Published:** 2026-04-11

**Authors:** Sho Hanai, Kiyoyuki Yanaka, Ken Akimoto, Hitoshi Aiyama, Nobuyuki Takahashi, Aiki Marushima, Eiichi Ishikawa

**Affiliations:** a Department of Neurosurgery, Tsukuba Memorial Hospital, 1187-299 Kaname, Tsukuba, Ibaraki 300-2622, Japan; b Department of Radiology, Tsukuba Memorial Hospital, 1187-299 Kaname, Tsukuba, Ibaraki 300-2622, Japan; c Department of Neurosurgery, Faculty of Medicine, University of Tsukuba, 2-1-1 Amakubo, Tsukuba, Ibaraki 305-8575, Japan

**Keywords:** Cerebral amyloid angiopathy, Cerebral microbleed, Cerebral small vessel disease

## Abstract

Cerebral microbleeds (CMBs) are commonly associated with cerebral small vessel disease (CSVD) and cerebral amyloid angiopathy (CAA) and usually present bilaterally. Unilateral distribution is rare and challenges current understanding of disease pathophysiology. We report the case of an asymmetric distribution of cerebral microbleeds. A 69-year-old hypertensive woman presented with acute right-sided hemiparesis and aphasia. Magnetic resonance imaging revealed an acute left frontal infarction, multiple lobar CMBs exclusively in the left hemisphere, and progressive left hemispheric atrophy without significant large-vessel stenosis. Although CAA and CAA-related inflammation (CAA-ri) were considered, definitive diagnosis was precluded by absent histopathological confirmation and incomplete imaging criteria. The case was considered within the spectrum of cerebral small vessel disease, acknowledging the diagnostic uncertainty. The patient was managed conservatively with antithrombotic therapy and strict blood pressure control, with no recurrent ischemic or hemorrhagic events. During 3-year follow-up, no recurrent ischemic or hemorrhagic events occurred; however, progressive cognitive function declined (MMSE: 20→16→6) and mild unilateral cerebral atrophy developed, suggesting ongoing small-vessel–related pathology. This case highlights the diagnostic challenges posed by asymmetric CMB distribution and underscores the importance of careful longitudinal clinical and imaging follow-up in patients with atypical CSVD presentations.

## Introduction

Cerebral microbleeds (CMBs) appear as small hypointense lesions on magnetic resonance imaging (MRI) and represent deposits of iron from blood breakdown products caused by underlying small vessel pathology [1,2]. They are commonly associated with cerebral small vessel disease (CSVD), including hypertensive arteriopathy and cerebral amyloid angiopathy (CAA). CMBs are linked to various conditions, including normal aging, Alzheimer’s disease, cerebral amyloid angiopathy (CAA), stroke, and traumatic brain injury [1,3,4]. Their prevalence increases with age [5] and vascular risk factors such as hypertension [6,7], diabetes mellitus [7], and prior stroke [7], and they are associated with an increased risk of both hemorrhagic and ischemic events [8]. Patients with isolated lobar microbleeds often share genetic and clinical risk factors as those with CAA-related intracerebral hemorrhage, suggesting severe CAA pathology [9].

CMBs are usually bilateral because cerebral small vessel disease (CSVD) typically involves the small blood vessels of both hemispheres [10]. However, in rare instances, CSVD can present with a predominantly or exclusively unilateral distribution. Such markedly asymmetric presentations are uncommon and raise diagnostic uncertainty, as they may reflect heterogeneous underlying mechanisms rather than a single disease entity. The potential causes of unilateral CSVD include diffuse axonal injury, neurovasculitis, cavernous malformations, cerebral autosomal dominant arteriopathy with subcortical infarcts and leukoencephalopathy (CADASIL), CAA-related inflammation (CAA-ri), and Parry–Romberg syndrome [[11], [12], [13]].

Here, we report a rare case of unilateral lobar CMBs accompanied by acute cerebral infarction and progressive hemispheric atrophy, highlighting the diagnostic and pathophysiological challenges posed by such atypical presentations. Careful longitudinal clinical and imaging follow-up is essential for understanding the mechanisms and consequences of unilateral small vessel disease.

## Case description

A 69-year-old woman was transferred to our hospital with acute-onset right-sided hemiparesis and aphasia. Before admission, the patient had been largely independent in activities of daily living and was able to manage household tasks such as gardening by herself. She had completed junior high school education and had no history of higher education. According to her family, she had experienced gradually progressive memory impairment for 2-3 years prior to admission, particularly difficulty remembering recent conversations and instructions. She had a history of hypertension; however, adherence to antihypertensive medication was inconsistent, and blood pressure control was poor. There was no notable family history of neurological disease, and she had no history of smoking or alcohol consumption.

On the day before admission, around noon, the patient developed dysarthria. Expecting spontaneous improvement, she did not seek medical attention. However, the symptoms persisted, and on the following day she developed right-sided weakness, prompting her to visit our outpatient clinic.

At admission, blood pressure was elevated at 186/84 mmHg. The patient’s level of consciousness was almost clear with a Glasgow Coma Scale score of 14 (E4V4M6). Neurological examination revealed right hemiparesis, slightly more severe in the upper extremity, and language disturbance including perseveration and word-finding difficulty. The mini-Mental State Examination score was 20/30 on arrival. Routine blood tests, including blood counts and coagulation profiles, were unremarkable (LDL cholesterol 109 mg/dL, HbA1c 5.8%, creatinine 0.77 mg/dL). Electrocardiogram revealed no evidence of arrhythmia, such as atrial fibrillation.

**Fig. 1:**
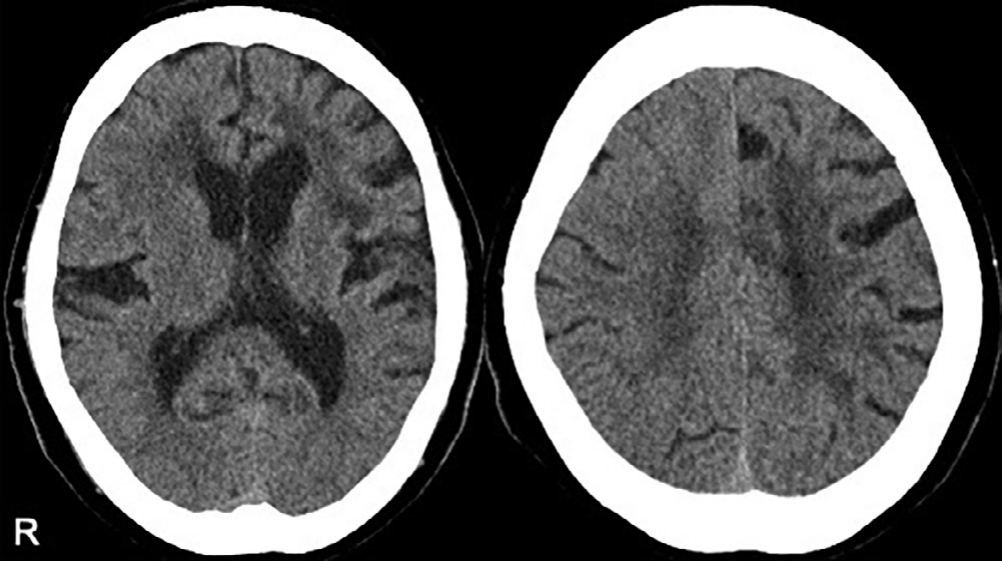
Non-contrast head CT obtained at initial presentation shows no definite acute hemorrhage or mass lesion. No clear established low-density area is identified at this time. This CT was performed as the initial screening examination prior to MRI. The letter ‘R’ indicates the right side of the patient in all images.

**Fig. 2:**
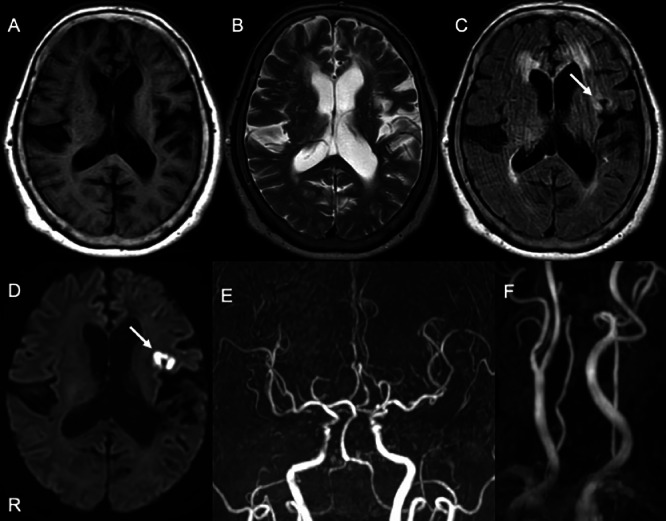
Magnetic resonance imaging (MRI) obtained at admission. (A) T1-weighted image, (B) T2-weighted image, (C) FLAIR image, (D) diffusion-weighted imaging (DWI), (E) intracranial magnetic resonance angiography (MRA), and (F) cervical MRA. DWI (D) demonstrates an acute ischemic infarction in the left frontal lobe (white arrow), which is also hyperintense on FLAIR (white arrow), indicating no DWI–FLAIR mismatch. (DWI was acquired using a single-shot echo-planar sequence; TR/TE = 4000/88 ms, b = 1000 s/mm²). FLAIR images show periventricular white matter hyperintensities and cerebral atrophy, more prominent in the left hemisphere. Both intracranial and cervical MRA reveal no significant stenosis or occlusion in the major cerebral or cervical arteries, and no lateralized vascular abnormalities. The letter ‘R’ indicates the right side of the patient in all images.

**Fig. 3:**
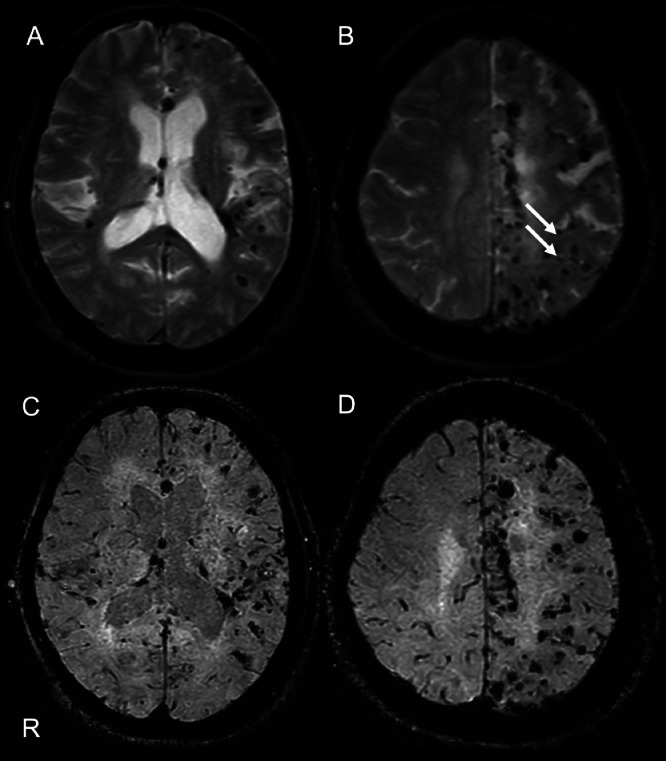
T2*-weighted imaging (T2*WI) (A,B) and susceptibility-weighted imaging (SWI) (C,D) demonstrating cerebral microbleeds (CMBs). Multiple hypointense foci consistent with CMBs are predominantly distributed in the left cerebral hemisphere (white arrows), while the right hemisphere shows only minimal involvement. SWI demonstrates findings similar to T2*WI, without additional lesions, confirming the markedly asymmetric, left-predominant distribution of microbleeds. The letter ‘R’ indicates the right side of the patient in all images.

Initial non-contrast brain CT showed no definite acute hemorrhage or mass lesion and no clear evidence of established infarction at the time of presentation (Fig. 1). MRI demonstrated a high-intensity lesion in the left frontal lobe on diffusion-weighted imaging (DWI; TR/TE = 4000/88 ms, b = 1000 s/mm²), which also showed hyperintensity on FLAIR imaging, indicating no DWI–FLAIR mismatch. These findings were consistent with an acute infarction in the left frontal lobe (Figs. 2A-D). Magnetic resonance angiography (MRA) revealed no significant stenosis in the cervical or the major cerebral arteries and no evidence of lateral vascular abnormalities (Figs. 2E and F). Carotid ultrasonography also revealed the absence of stenosis in the cervical carotid arteries. The infarction was therefore diagnosed as likely due to atherosclerotic disease. Fluid-attenuated inversion recovery (FLAIR) imaging revealed bilateral periventricular hyperintensities and cerebral atrophy, which were more pronounced on the left side. T2*-weighted imaging (T2*WI) revealed several CMBs, predominantly localized to the left hemisphere. Susceptibility-weighted imaging (SWI) was also performed and demonstrated a distribution of microbleeds similar to that seen on T2*-weighted imaging, without additional lesions or new findings (Fig. 3). Gradient-recalled echo (GRE) sequences were not obtained in this case.

The acute ischemic stroke was managed conservatively with intravenous hydration, antiplatelet therapy (intravenous ozagrel sodium for two weeks followed by oral cilostazol 100 mg twice daily), and antihypertensive therapy with an oral calcium channel blocker. The patient underwent rehabilitation therapy during hospitalization. The patient’s hemiparesis and aphasia gradually improved with rehabilitation.

**Fig. 4:**
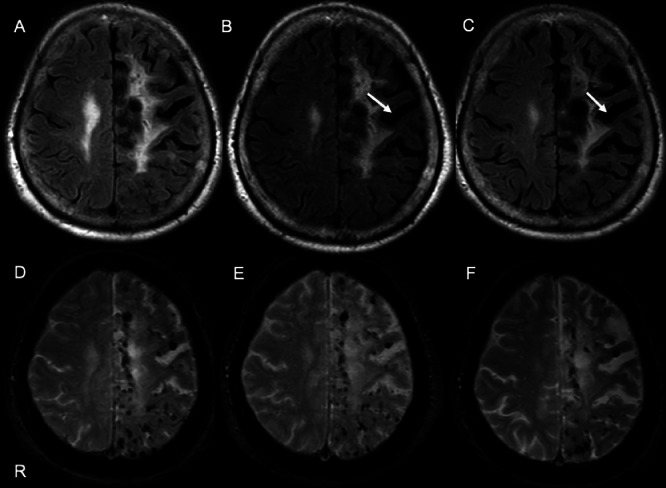
Fluid-attenuated inversion recovery (FLAIR) images obtained at initial presentation (A), 1-year follow-up (B), and 3-year follow-up (C) demonstrate gradual enlargement of the left cerebral sulci (white arrow), indicating progressive left hemispheric atrophy over time. T2*-weighted images obtained at the same time points (D–F) show multiple cerebral microbleeds predominantly distributed in the left hemisphere, with no increase in number or change in spatial distribution during follow-up. These findings indicate that structural atrophy progressed independently of microbleed burden. The letter ‘R’ indicates the right side of the patient in all images.

Upon discharge, the patient continued outpatient management with antithrombotic therapy for secondary stroke prevention and strict blood pressure control because of the presence of multiple CMBs. During follow-up, cognitive function showed a gradual decline over time. At approximately 2 years after onset, the MMSE score decreased slightly to 16/30 from 20/30 at admission; however, daily functioning remained largely preserved, and this change was not considered clinically significant at that time. In contrast, at the most recent evaluation 3 years after onset, the MMSE score had declined markedly to 6/30, indicating a substantial progression of cognitive impairment. During this period, no recurrent ischemic or hemorrhagic events occurred, and serial MRI demonstrated mild progression of left hemispheric atrophy without an increase in the number or distribution of cerebral microbleeds (Fig. 4).

Although the MMSE alone cannot fully characterize domain-specific cognitive decline, the progressive reduction in scores suggests ongoing vascular and/or neurodegenerative cognitive impairment. No recurrent ischemic or hemorrhagic events were observed during follow-up.

## Discussion

CMBs are small perivascular deposits of hemosiderin that reflect prior microhemorrhages or localized vessel wall damage and a key imaging marker of CSVD [4,10]. In hypertensive vasculopathy, CMBs predominantly involve deep brain regions such as the basal ganglia, thalamus, brainstem, and cerebellum, whereas in CAA they are typically located in the cerebral lobes [10]. These distinct distributions reflect differences in the affected vascular territories, with hypertensive arteriopathy involving deep penetrating arteries and CAA affecting cortical and leptomeningeal vessels, leading to amyloid accumulation, inflammation, and oxidative stress [11].

CSVD is an umbrella term encompassing disorders of small cerebral arteries and microvessels, including lacunar infarction, white matter hyperintensities, enlarged perivascular spaces, and CMBs [14,15]. Although CSVD typically affects both hemispheres, resulting in bilateral imaging abnormalities, markedly asymmetric or unilateral presentations are exceptionally rare [16]. To date, only a limited number of unilateral CMB cases have been reported, most of which are associated with CAA rather than hypertensive arteriopathy [11]. Such atypical distributions pose diagnostic challenges, as the Boston criteria for MRI-based diagnosis of CAA do not explicitly address markedly asymmetric or unilateral CMB patterns [17].

Multiple mechanisms may underlie the asymmetric distribution of CSVD-related pathology. One plausible factor is the presence of undetected microvascular abnormalities causing focal arteriolar stenosis or occlusion leading to reducing perfusion and increasing susceptibility to CSVD-related damage in one hemisphere [7]. Another potential explanation involves localized hemodynamic dysregulation. Although generalized factors, such as prolonged hypertensive stress, are well-known contributors to CSVD, localized variations in perfusion or vascular reactivity may result in asymmetric damage [8]. Localized vascular inflammation or immune responses may lead to asymmetric endothelial dysfunction and increased vascular permeability [4]. Considering the suspected involvement of both CAA and CSVD in this patient, uneven amyloid deposition or regional vascular inflammation may have contributed to the predominance of CMBs in the left hemisphere. In addition to vascular and inflammatory factors, systemic embolic events may also help explain the unilateral presentation of CSVD. Conditions, such as atrial fibrillation, can produce microemboli that preferentially affect one cerebral hemisphere, causing localized ischemia and potentially triggering CSVD-related changes [9]. Furthermore, genetic predisposition may influence susceptibility to unilateral CSVD. Some genetic conditions—such as Parry–Romberg syndrome—have been linked to asymmetric cerebrovascular abnormalities [13]. The diagnosis of CAA was carefully re-evaluated in this case using the Boston criteria v2.0 [17]. The patient demonstrated multiple strictly lobar cerebral microbleeds, which represents one imaging marker consistent with CAA. However, other key criteria required for a diagnosis of probable CAA were not fulfilled. Specifically, there was no history of spontaneous lobar intracerebral hemorrhage, no evidence of cortical superficial siderosis, and no characteristic transient focal neurological episodes. Accordingly, this case could be classified at most as possible CAA under the Boston criteria v2.0. Importantly, the highly unilateral distribution of microbleeds, progressive hemispheric atrophy, and coexistence of ischemic infarction represent atypical features that are not fully captured by current CAA diagnostic criteria. These limitations highlight the difficulty of definitive classification in cases with markedly asymmetric presentations.

To contextualize the present case within the spectrum of asymmetric cerebral microbleeds, we summarized previously reported unilateral or asymmetric CMB cases in Table 1. This includes cases suspected to involve cerebral amyloid angiopathy (CAA), CAA-related inflammation (CAA-ri), and Parry–Romberg syndrome. In the case reported by Wong et al. [13] a 30-year-old woman with Parry–Romberg syndrome demonstrated innumerable susceptibility foci throughout the right cerebral hemisphere on SWI, indicating unilateral microbleeds without a clear CAA diagnosis. Compared with these reports, our case uniquely combines unilateral lobar microbleeds with an acute infarction and long-term progressive hemispheric atrophy, supporting the notion of a complex small vessel disease spectrum.

**Table 1:** Summary of previously reported cases involving unilateral or asymmetric cerebral microbleeds or related imaging findings.

Year	Author	Age/Sex	Diagnosis	Distribution of CMBs	Associated lesions	Follow-up /Outcome
2015	Wong et al. [13]	30/F	Parry–Romberg syndrome	Unilateral (right hemisphere)	Facial hemiatrophy; no infarction	Stable imaging findings
2023	Sokola et al. [26]	71/F	Probable CAA	Markedly asymmetric lobar	Lobar hemorrhage	Recurrent events
2024	Romero et al. [27]	52/M	Probable CAA	Asymmetric lobar	Lobar hemorrhage	Recurrent events
2024	Manorenj et al. [11]	75/F	Likely CAA / hypertensive microangiopathy	Right hemispheric microbleeds	Lobular cortex & deep atrophy periventricular hyperintensity	Recovery
2026	Present case	69/F	CSVD (possible CAA)	Unilateral lobar	Ischemic infarction, progressive atrophy	Cognitive decline over 3 years

Abbreviations: CAA, cerebral amyloid angiopathy; CMBs, cerebral microbleeds; CSVD, cerebral small vessel disease.

In addition to the previously mentioned differential diagnoses, CAA-ri should be considered [12]. Although CAA-related inflammation (CAA-ri) was considered as a differential diagnosis, several features argued against it as the primary cause of the patient’s symptoms. Specifically, there was no subacute encephalopathy, no prominent asymmetric vasogenic edema on FLAIR imaging, no evidence of steroid responsiveness, and no cerebrospinal fluid analysis suggestive of inflammation, all of which are commonly observed in typical CAA-ri. Unlike typical CAA, which typically presents with bilateral lesion distribution, CAA-ri may cause localized inflammation, potentially leading to asymmetric lesions, as observed in this case. Moreover, CAA-ri is associated with an increased risk of thrombus formation due to vascular inflammation, which could account for the acute cerebral infarction observed in this patient. In typical cases of CAA-ri, asymmetric white matter edema on FLAIR images is often more pronounced; however, this was not evident in our case. Diagnosis of CAA-ri requires assessment of clinical symptoms, imaging findings, cerebrospinal fluid analysis (including elevated inflammatory markers), and, if possible, pathological confirmation *via* biopsy. This case raises the possibility of underlying CAA-related pathology; however, available evidence was insufficient to establish a definitive diagnosis of CAA or CAA-ri.

The progressive cognitive decline (MMSE: 20→16→6 over 3 years) observed during long-term follow-up accompanied by unilateral atrophy is an important finding in this case. Although MMSE alone cannot fully characterize domain-specific cognitive decline, the progressive reduction suggests ongoing small-vessel–related neurodegeneration. Such a course is compatible with chronic CAA-related pathology or asymmetric CSVD, even in the absence of histopathological confirmation. This observation supports the concept that unilateral microvascular pathology can have long-term cognitive consequences.

Currently, there is no specific treatment for CSVD; however, blood pressure regulation is the most critical intervention. Lowering blood pressure reduces the risk of cerebral infarction, intracranial hemorrhage, and white matter hyperintensities [18,19]. Brain atrophy is associated with white matter hyperintensities [20], maintaining optimal blood pressure levels may also help prevent progressive brain atrophy. In cases of CAA, although hypertension does not significantly influence the age of first intracranial hemorrhage or its recurrence [21], it has been shown to increase the overall incidence of intracranial hemorrhage [22]. Therefore, blood pressure control remains a key strategy for mitigating CAA-related pathology [23].

Blood pressure management was a central component of treatment, with a target systolic blood pressure below 140 mmHg, achieved using oral calcium channel blockers and regular outpatient monitoring. Strict blood pressure control was maintained throughout follow-up to reduce the risk of both ischemic and hemorrhagic complications associated with small vessel disease. In our case, antithrombotic therapy was administered to prevent recurrent ischemic stroke. The use of antithrombotic agents is associated with an increased risk of CMBs [24], the immediate risk of ischemic stroke recurrence in the acute phase often outweighs the potential hemorrhagic complications [25]. However, in patients with multiple CMBs, particularly those with more than five lesions, the long-term risk of hemorrhagic events may be equivalent to the risk of recurrent ischemic stroke [25]. Further studies are necessary to determine the optimal balance between ischemic and hemorrhagic risks in patients with multiple CMBs.

## Conclusion

CSVD includes a spectrum disorders such as arteriolosclerosis and cerebral amyloid angiopathy, all of which affect the brain’s small vessels. Due to its diverse clinical manifestations and imaging patterns, CSVD can be particularly difficult to diagnose and manage—particularly in atypical cases, such as unilateral presentations. Although the precise mechanisms underlying asymmetric CSVD remain poorly understood, this case illustrates that unilateral CMBs may represent a distinct and underrecognized manifestation within the CSVD spectrum, with potential long-term cognitive consequences. In the absence of histopathological confirmation, a CSVD-based framework provides a practical and inclusive diagnostic approach for such diagnostically ambiguous cases. Effective blood pressure control is a key strategy for preventing disease progression and mitigating both the clinical symptoms and neuroimaging changes associated with CSVD. Further research is essential to explore and develop targeted therapeutic strategies for patients with atypical CSVD presentations.

## Patient consent

A written informed consent was obtained from the patient for the publication of this case report.
